# PHTFNet-RPM: a probabilistic hybrid network with RPM for tobacco root disease forecasting

**DOI:** 10.3389/fdata.2025.1705587

**Published:** 2025-11-10

**Authors:** Yunhong Bu, Tingshan Yao, Shaowu Geng, Renjie Huang

**Affiliations:** 1 Chuxiong Company of Yunnan Provincial Tobacco Corporation, Chuxiong, China; 2 National Engineering Research Center for Citrus Technology, Citrus Research Institute, Southwest University, Chongqing, China; 3 College of Computer and Information Science, Southwest University, Chongqing, China

**Keywords:** hybrid neural network, random period mask, uncertainty estimate, plant disease forecasting, time-series modeling, smart agriculture

## Abstract

**Introduction:**

Tobacco growers usually face particular challenges in predicting the risks of tobacco root diseases due to complex pathogenesis, concealed early symptoms, and heterogeneous farm conditions.

**Methods:**

To address this problem, we proposed a flexible Probabilistic Hybrid Temporal Fusion Network with Random Period Mask (PHTFNet-RPM). This model is designed to forecast future multi-day disease incidences and indices. It incorporates a hybrid input structure with RPM to handle configurable static management variables and time-series data of weather factors and disease metrics, using the RPM to simulate diverse absences of historical observations. The model's internal hierarchically aggregated modules learn cross-variable and cross-temporal feature representations to model the complex non-linear relationships. Furthermore, probabilistic theory-based uncertainty quantification is designed to enhance the model's credibility and reliability.

**Results:**

The proposed PHTFNet-RPM was validated using a large-scale time-series dataset of tobacco root diseases, organized from 20-year meteorological and disease survey records in Chuxiong Prefecture, Yunnan Province. Extensive comparative experiments demonstrated that our model achieves a 4.44%–16.43% lower mean absolute error (MAE) than existing models (including LR, SVR, CNN-LSTM, and LSTM-Attention).

**Discussion:**

The results confirm that the model can reliably forecast disease progression trends under different configurations, even when relying solely on historical weather observations. The integration of uncertainty quantification provides a robust tool for assessing prediction reliability, offering significant practical value for disease management.

## 1 Introduction

Tobacco plant is an important cash crop in China, with an output value of approximately 600 billion yuan. In tobacco cultivation, it is critical to manage diseases because they will cause a decline in yield and quality of tobacco leaf. In China, more than 20 common diseases damage tobacco crops. Among them, root diseases are highly transmissible, severe in impact, and difficult to control (Nyvall, 2013). For example, for two root diseases of black shank and black root rot, when inspecting tobacco plants' health statuses and confirming diseases, the plants have been severely harmed and it is difficult to cure them because they have latent period and the root damages can not be easily found (Pandey, 2023). This significantly affects tobacco yield and quality. To prevent these diseases, the scientific method is to monitor growing conditions, predict the disease outbreak risks and implement some management measures to clear pathogens and block pathogen transmission. Accordingly, it is key to predict disease outbreak and severity according to the growing condition and management levels (Patil et al., 2022; Dharanya et al., 2025; Chen et al., 2025).

However, the causes of tobacco root diseases are complex and it is difficult for prediction and early-warning of disease outbreak. The influence factors contain climatic condition, variety selection, greenhouse seedling management, soil condition, fertilization practices and field management (Kishan Das Menon et al., 2021). Taking black shank for example, high temperature and humidity are the favorable conditions in which it easily outbreaks. Besides the historical meteorological factors, the conditions conducive to disease onset and spread also rely on soil drainage and scientific management measures (Csinos, 1999). Totally, these influence factors interact on each other, contributing more or less to the disease outbreak and development. For accurate prediction and early-warning of tobacco root diseases, it is necessary to design reasonable quantification of each factor and further model the relation these influence factors and disease risks (Suarez et al., 2023).

To date, many researches have been done for reducing and controlling tobacco root diseases in agriculture and in the artificial intelligence (AI) domain. Agronomists study the causes and suppression methods of root diseases and further develop biocides and farm chemicals (Qian et al., 2019; Zhu et al., 2024). AI scientists focus on analyzing the relations between tobacco plants' growing conditions and root diseases and automatically diagnosing and forecasting diseases (Rao et al., 2016; Aldaour and Abu-Naser, 2019; Cai et al., 2019; Chin et al., 2022; Dharanya et al., 2025; Chen et al., 2025). They collect sensor data to describe the growth conditions, including the soil moisture and nutrient status, and meteorological factors such as temperature, humidity, illuminance and cloud cover (Kishan Das Menon et al., 2021). Based on the sampled sensor data, prediction models are built to estimate the level of disease risk or the detailed technological parameters, for instance, the disease incidence and the disease index. Furthermore, some prevention measures can be automatically recommended according to the estimated disease risk levels and diagnosis results.

Especially, in the recent years, the significant advancements in AI and machine learning technologies (Delfani et al., 2024) have greatly propelled the development of tobacco disease risk prediction. The representative works mainly focus on two aspects of the intelligent diagnosis and the risk prediction. In the diagnostic researches, the expert system (Rao et al., 2016) and the Computer Vision (CV) (Sun et al., 2024) are the mainstream technologies. The description of lesions is fed into the intelligent system constructed by expert experiences (knowledge base and inference rules) (Aldaour and Abu-Naser, 2019). It automatically derives the categories and severities of diseases. In the CV-based diagnostic methods, the images of tobacco plants are sampled and the lesion features in the images will be automatically extracted through the hand-crafted feature descriptors or the learned feature extractors (Xu et al., 2022). The extracted lesion features will be combined with the classification and regression technologies, e.g. the Support Vector Machine (SVM) and Deep Neural Networks (DNN) (Lin et al., 2022), to recognize and analyze the diseases. However, the intelligent diagnosis technologies are less effective to provide an early-warning of tobacco root diseases because their early lesions are invisible under the earth.

Rather than relying on the analysis of lesions on tobacco plants, the risk prediction mainly utilizes the growth conditions and disease-resistant characteristics of crop varieties to estimate the likelihood of disease outbreak (González-Doḿınguez et al., 2023). The disease risk prediction models can be mainly classified into three categories: the data-based ones, the process-based ones, and time-series forecasting ones. Data-based models explore the prediction rules and mechanism by analyzing the relations between the host plant, the pathogen and the environment, using statistical tools and AI algorithms together with a set of observed data (Cai et al., 2019; Van Nguyen, 2021). For a data-based prediction model, the forecasting accuracy mainly relies on the quantity and quality of the historical data about disease outbreak. It is partly affected by the modeling methods and learning algorithms. However, in most cases of forecasting crops diseases, the historical observed records are scarce and precious. Process-based models construct the parameterized functions driven by environmental variables to describe the change process of disease in space and/or time (Salotti et al., 2022; Patel et al., 2020). For a process-based model, specific expert knowledge is required and the constructed model has less generalization, which generally applies to a single kind of disease.

Time series forecasting models integrate the characteristics of the data-based models and the process-based models. Multiple methods, such as smoothing methods, decomposition methods, regression methods, and DNN, can be utilized to designed the time series models (Masini et al., 2023). To optimize them, time-series data, such as continuously varying historical weather records and growth conditions, is required. The model automatically learn the relation between disease risks and crops' temporally continuous growth conditions (historical weather) from the time-series data. Moreover, the varying process of disease development can be implicitly exhibited because time series data training the models contains such varying process (Wang et al., 2024; Chen et al., 2025). However, compared to the data-based and process-based models, time series forecasting models impose stricter requirements on data samples, necessitating the measurement of continuous disease progression. It implies significantly higher costs (González-Doḿınguez et al., 2023).

Recently, crop disease forecasting based on time series models and weather factors has attracted more attention due to the development of agricultural big data and the successful application of time series models in the fields such as energy, retail and economics (Masini et al., 2023). For example, Krishna and Prema (2023) experimentally analyzed multiple time series models such as ARIMA, MLP, RNN and LSTM in predicting fruit rot disease incidence. Chen et al. (2024) compared three models such as LSTM, LSTM-Attention and Transformer-based Informer (Zhou et al., 2021) in forecasting the incidence of pests in tea gardens. They revealed that the LSTM-Attention model integrating sequence-to-sequence encodings and the attention mechanism can better learn the temporally-related features. Wang and Li (2025) integrated ARIMA and deep learning to propose a ARIMA-LSTM model for pest and disease prediction and management of sugarcane. Multiple hybrid models combining CNN and LSTM (Wang et al., 2024; Guo et al., 2024; Chen et al., 2025) are proposed to forecast different crop diseases. In forecasting tobacco diseases, Chen et al. (2025) proposed a CNN-LSTM model to forecast four tobacco diseases by utilizing time-varying meteorological factors.

By reviewing the previous literatures about forecasting crop diseases, we find that the following problems are available in the current researches. Firstly, most forecasting models are constructed based on meteorological factors, but the field management information is less considered (Cai et al., 2019; Van Nguyen, 2021; Patel et al., 2020; Chin et al., 2022; Chen et al., 2025). However, besides meteorological factors, the tobacco root disease development is severely affected by field quality and management factors such as soil drainage, variety resistance and management level (Suarez et al., 2023). If these factors are not integrated into a forecasting model, it will causes that the model can not adaptively adjust the forecasting function according to these related factors. Especially, for most growers in a tobacco producing area, the meteorological factors are similar, but they have diverse field conditions and management levels, each tobacco field may have a different disease risk profile. To better predict the disease risks in each tobacco field, the information of land quality and field management should be quantified and embedded into the model besides the time-varying meteorological factors. Accordingly, the model user can flexibly configure the model according to his land information and management level, so that the model can adaptively adjust its forecasting functions.

Secondly, in the domain of forecasting crop diseases (González-Doḿınguez et al., 2023), most existing models are unable to identify data quality and provide reliable uncertainty approximation. This results in a lack of interpretability in reasoning and forecasting. Namely, the data uncertainty, model uncertainty and predictive uncertainty are not considered and Bayesian learning theories are not introduced in modeling (Zhou et al., 2022; Wang and Yeung, 2020). A model integrated the data uncertainty estimation can better process the noise data, which is usually caused by the irreducible error in the observation process. For example, the lesions is possibly measured wrongly and the disease index could be computed in error (Pandey, 2023). If a model integrates the model uncertainty and predictive uncertainty, it can effectively emulate the uncertainty that experts face when making predictions. This helps the model provide reliable and interpretable predictions. In other words, given an observation record, it not only predicts the disease risk but also estimates the reliability of the prediction using the learned model, which helps model users to make scientific disease managements.

Finally, less crop disease forecasting models considered the diverse absences of disease records in designing and training models (González-Doḿınguez et al., 2023; Masini et al., 2023), which causes a significant degradation of forecasting precision when lacking recent historical disease records and mainly relying on meteorological factors. However, historical disease records are not updated in a timely manner and the diverse absences of them are frequently encountered situations because the assessment and measurement of disease severity are time-consuming processes. Most existing models assumed that all historical disease records are available and training data usually can not cover all situations of diverse absences of disease records (Chen et al., 2024; Wang et al., 2024; Guo et al., 2024; Chen et al., 2025). When training the time series forecasting models, the model's predictive learning will depend more heavily on historical disease data than on meteorological variables. In forecasting, when lacking the recent historical disease records, the forecasting precision will decrease severely. Therefore, in modeling or designing learning algorithms, it is necessary to simulate the diverse absences of disease records in real-world application scenarios so that the model can learn how to adaptively balance the historical disease records and meteorological variables to make prediction.

Based on the above observed problems, we proposed a flexible Probatilistic Hybrid Temporal Fusion Network with Random Period Mask (PHTFNet-RPM) for forecasting tobacco root diseases based on the architecture of temporal fusion transformer (Lim et al., 2021). The PHTFNet-RPM forecasting model mainly consists of condition-configurable hybrid-structured input with RPM, multi-level time-series feature extraction and fusion, and multi-horizon multi-target prediction. Moreover, the reliability estimate of learned model and forecasted results is integrated in model design and model learning by the Monte Carlo Dropout-based Bayesian inference (Sankararaman et al., 2022) and the predictive confidence interval (Cai, 2002). To validate the proposed model, we constructed a time-series dataset of tobacco root disease and conducted extensive contrast experiments. The experimental results show our approach significantly outperforms the recent methods, e.g., the CNN-LSTM for forecasting tobacco diseases (Chen et al., 2025) and the LSTM-Attention for predicting insect pests in tea gardens (Chen et al., 2024). Furthermore, the detailed model analysis experiments validate that RPM enables our model to extract effective features for accurately forecasting the disease progression trend, with minimal impact from diverse absences of disease data.

Our contributions are concluded as following: (1) The proposed PHTFNet-RPM model integrates the flexible hybrid-structured input and the powerful representational capacity to conduct the condition-adaptive prediction. Its hybrid-structured input with RPM can well incorporate the time-series historical weather and disease observations together with configurable field management conditions. Moreover, RPM simulates the diverse absences of historical data. The model's hierarchically aggregated modules collectively achieve powerful adaptive feature representation. (2) The uncertainty quantification of forecast results enhances prediction credibility and interpretability by providing probabilistic bounds, enabling risk-aware decision-making. The uncertainty estimate of learned model ensures model robustness and reliability in real-world deployments. (3) We constructed the first large-scale time series dataset for forecasting tobacco root diseases, including black shank and black root rot. It contains about 12 thousands of time-series samples from real-field meteorological and disease records for 20 years, from 2003 to 2022, covering disease survey records in the field management period of tobacco from transplanting to harvest.

## 2 Materials and data processing

### 2.1 Collection of tobacco root disease data and weather data

This paper takes management data of tobacco root diseases from Chuxiong Company of Yunnan Provincial Tobacco Corporation as the survey and research subjects. The data collection lasted for 20 years (from 2003 to 2022) and the data sources cover nine counties and districts in Chuxiong Prefecture. The collected data mainly includes three categories: disease management data closely-related to observation stations, continuously-recorded disease data and historical daily weather records as shown in Table 1. In collecting disease records in each year, the disease surveys were conducted every 5 or 10 days from transplanting to the field until tobacco leaf harvesting. In the disease surveys, a five-point sampling method (Pandey, 2023) was used to evaluate disease incidence and disease index, with 50 plants surveyed at each point, resulting in a total of 250 plants surveyed per field. According to the disease statuses of the investigated 250 tobacco plants, the disease incidence *y*_*inc*_ and the disease index *y*_*ind*_ are manually computed as follows:


$$\begin{array}{l} y_{inc}=\frac{m}{n}, \end{array}$$ (1)

$$\begin{array}{l} y_{ind}=\frac{\overset{r}{\underset{i=1}{\sum }}s_{i}*n_{i}}{n*s_{r}}. \end{array}$$ (2)

Here, *n* is the total number of investigated plants, *m* is the number of infected plants. The severity of disease is classified into *r* grades. *s*_*i*_ corresponds to the score of the *i*-th grade. *n*_*i*_ is the number of plants which are identified as being with the *i*-th grade disease. *s*_*r*_ is the maximum score, which corresponds to the most serious disease. *y*_*inc*_ and *y*_*ind*_ respect the prevalence and severity of tobacco root diseases.

**Table 1:** Variable description in disease data.

**Variable name**	**Variable type**	**Variable value**	**Description of variable and its value**
**Independent variables (static, field management)**			
Survey year	Discrete	{1, ⋯ , *n*_*y*_}	1 and *n*_*y*_ individually indicate the starting and ending year.
Survey location	Discrete	{1, ⋯ , 9}	Nine numbered county-level survey stations.
Disease category	Discrete	{1, 2}	Two categories of diseases: black shank and black root rot.
Tobacco variety	Discrete	{1, 2, 3, 4}	Four varieties related to disease resistance.
Rotation crops	Discrete	{1, ⋯ , 6}	Six rotation crops related to soil pathogen load.
Seedling means	Discrete	{1, ⋯ , 5}	Five seedling means related to pathogen load on plants.
Management level	Discrete	{1, ⋯ , 5}	Five grades of disease precaution and control.
Past prevalence	Discrete	{1, ⋯ , 5}	Historical disease outbreak scale.
**Independent variables (time-varying, weather condition)**			
Sampling date	Discrete	[1, ⋯ , *n*_*d*_]	1 and *n*_*d*_ individually indicate the starting and ending dates.
Max temperature	Continuous	[15.0, 40.0]	It facilitates pathogen transmission.
Avg temperature	Continuous	[10.0, 30.0]	Same to the above.
Relative humidity	Continuous	[0.0, 100.0]	Same to the above.
Daily Rainfall	Continuous	[0.0, 1000.0]	Affecting soil and air humidity.
Sunshine hours	Continuous	[0.0, 14.0]	Affecting soil and air humidity.
Wind speed	Continuous	[0.0, 10.0]	Affecting soil and air humidity.
**Target variables (time-varying metrics)**			
Disease incidence	Continuous	[0.0, 1.0]	It describes disease prevalence.
Disease index	Continuous	[0.0, 1.0]	It describes disease severity.

### 2.2 Data processing

**Figure 1:**
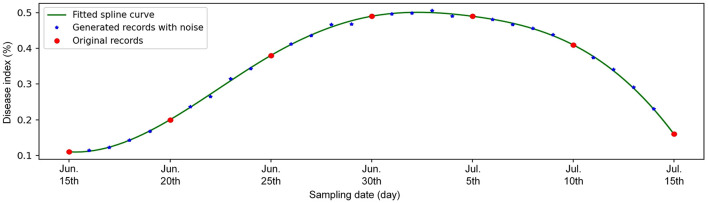
Generation of daily disease index records by applying the spline interpolation method on historical records with the intervals of 5 days.

In the original management data, sampling intervals of historical disease records possibly are inconsistent, e.g. every 5 days or every 10 days in different years as shown in Figure 1. Moreover, the disease records are not related to the weather data because the disease data and the weather data are collected individually. Therefore, it is necessary to process and integrate them for better training the forecasting model.

#### 2.2.1 Complement and argumentation of disease historical records

In order to keep sampling intervals consistent and increase data quantity, we adopt the spline interpolation method to argument disease records with multi-day interval as daily records. As shown in the Figure 1, given the original seven records of disease index of black shank (recording once every 5 days from June 15th to July 15th in 2014) at the Yaoan survey station, a cubic spline function is first obtained. Then, for the dates without practical records, their disease index values are sampled on the fitted spline function. To simulate the manual errors, the new generated values are added by a Gaussian noise. In this way, the daily disease index will be obtained.

#### 2.2.2 Integration of disease and weather data for dataset construction

The disease forecasting model aims to construct the mapping relation between independent variables (static management information and time-series weather data) and target (dependent) variables as shown in Table 1. For the data-driven learning model, recorded data for independent and target variables should be fed into the model for optimizing model parameters. We integrated the heterogeneous data and organized all records as the JSON-formatted dataset according to the literature (Lim et al., 2021) and the Pytorch Forecasting package.^1^ We first integrated the heterogeneous data into individual time-series entities using composite keys of year, station, and disease. Each entity has hybrid-structured data as shown in Table 1, i.e. static agronomic variables (vector), meteorological time-series (matrix, a dimension denotes date, the other dimensions indicate weather variables), and pathological time-series (matrix, a dimension denotes date, the other dimensions indicate disease metrics), where the date spans from transplanting to harvest. After configuring historical and future window sizes, by decomposing meteorological time-series and pathological time-series, each entity can be decomposed as multiple training samples.

## 3 Methodology

### 3.1 Overview

**Figure 2:**
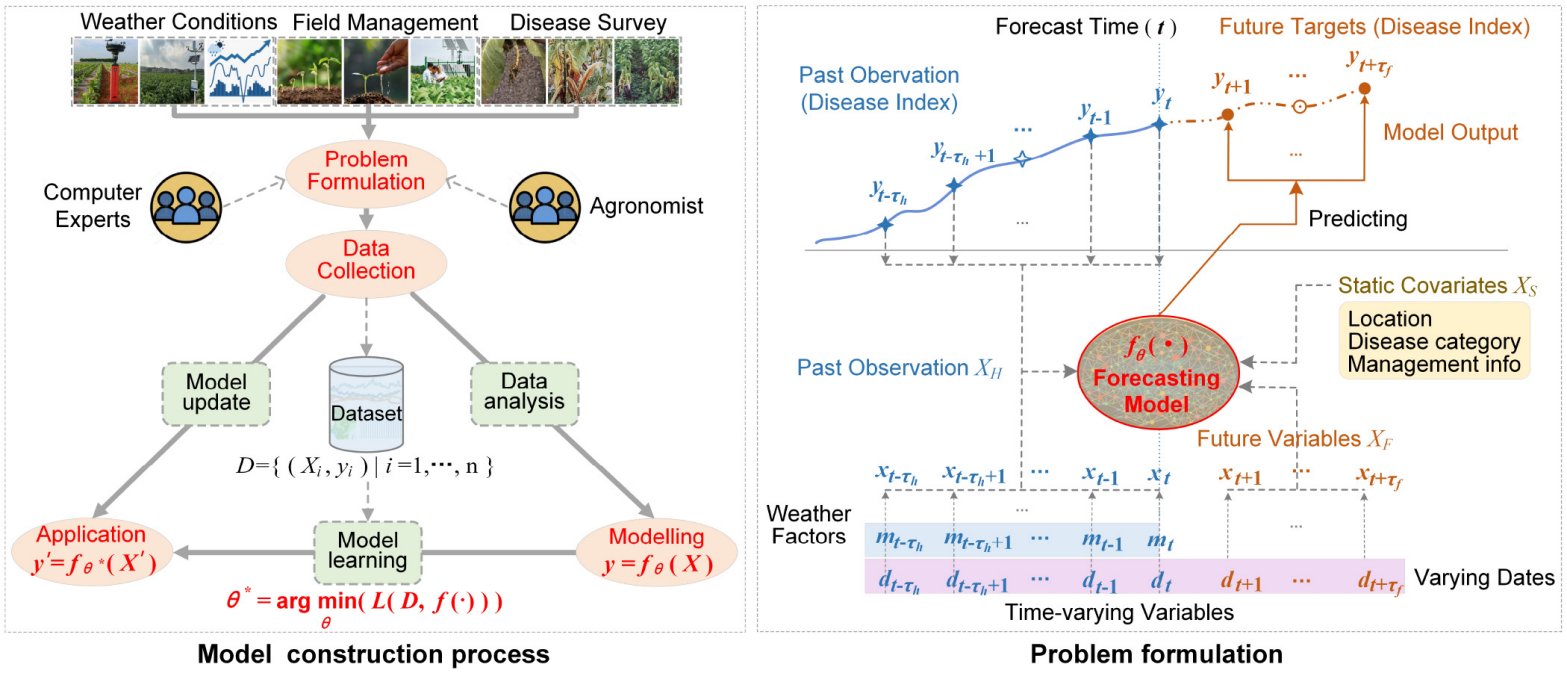
Model construction process and problem formulation for forecasting tobacco diseases. When predicting multiple target variables, there will be multiple predicted curves. Here, for simplicity, only the disease index is shown.

According to the recent researches on forecasting crop diseases (Chen et al., 2025; Wang et al., 2024; Chen et al., 2020), we adopted a four-stage paradigm for constructing and deploying the disease forecasting model as shown in Figure 2. (1) Problem formulation. The independent variables (model input *X*) and forecast targets (model output *y*) are firstly determined by analyzing this forecast task. (2) Dataset construction. Based on the variables in model's input *X* and output *y*, by data collection, the dataset *D* = {(*X*_*i*_, *y*_*i*_)|*i* = 1, ⋯ , *n*} can be constructed according to Section 2. (3) Modeling and optimizing. By investigating the complex relationships between the independent variables in *X* and the target variables in *y*, a suitable modeling technology is selected to design a parameterized function *f*_θ_(·) for representing the relationship between *X* and *y*. Then, by designing the loss function *L*(·) and learning algorithm, the model parameter θ can be optimized as $\theta^{*}=\underset{\theta }{\arg\min}\underset{\left(X_{i},\;y_{i}\right)\in D}{\sum }L\left(f_{\theta }\left(X_{i}\right),y_{i}\right)$. The related contents will be represented in detail in Sections 3.2 and 3.3. (4) Model deployment and maintenance. Based on the learned model $f_{\theta^{*}}\left(\cdot \right)$ with the optimal parameter θ^*^, for any new observation *X*′, the target can be predicted as $y^{\prime }=f_{\theta^{*}}\left(X^{\prime }\right)$. Especially, when collecting enough new data, the model $f_{\theta^{*}}\left(\cdot \right)$ can be fine-tuned.

Among the four stages, the problem formulation is the primary preliminary step, as it dictates the modeling approach and the form of the model's inputs and outputs. In this work, we formulated the forecast task as the time-series model *y* = *f*_θ_(*X*) as shown by the problem formulation in Figure 2 according to the literature (Lim et al., 2021). Here, θ denotes the learnable model parameter. The model input *X* = {*X*_*H*_, *X*_*F*_, *X*_*S*_} is the hybrid-structured data. The time-series matrix $X_{H}\in {\mathbb{R}}^{n_{h}\times \left(\tau_{h}+1\right)}$ denotes the historical observation, where τ_*h*_ indicates the size of historical time window, *n*_*h*_ is the number of observed variables including the weather factors, date and disease risk metrics. The time-series matrix $X_{F}\in {\mathbb{R}}^{n_{f}\times \tau_{f}}$ denotes the future known data, where τ_*f*_ indicates the size of future time window, *n*_*f*_ is the number of prior-known variables such as date and the others. $X_{S}\in {\mathbb{R}}^{n_{s}}$ denotes the static covariates, which indicates the configurable prediction conditions, where *n*_*s*_ indicate the number of static variables as shown in Table 1. The model output $y\in {\mathbb{R}}^{n_{m}\times \tau_{f}}$ indicates the multi-target and multi-horizon predicted result, where *n*_*m*_ denotes the number of disease metrics (target variables). For instance, *y*(*j, k*) indicates the predicted value of *j*-th target variable on the *k*-th day, where 1 ≤ *j* ≤ *n*_*m*_ and *t*+1 ≤ *k* ≤ *t*+τ_*f*_.

### 3.2 PHTFNet-RPM based forecasting model

#### 3.2.1 Networks architecture

Based on the analysis on tobacco root disease epidemiology and collected disease data, we design Deep Neural Networks (DNN) to model the forecasting model *f*_θ_(·). Moreover, the designed DNN should meet the following requirements: (1) It can be fed hybrid-structured data including continuous time-varying historical observation *X*_*H*_, continuous future prior-known variables *X*_*F*_ and discrete time-invariable (static) variables *X*_*S*_ about field management information. (2) It can express flexible complexities by configuring varied model parameters and prediction conditions, so that it adapts to data-scale and problem complexity, and adaptively adjusts the forecasting capability in different conditions of field management. (3) It can provide uncertainty estimate on predicted results to simulate the reasoning process of human experts making a fuzzy forecast. The uncertainty is caused by the possibly inaccurate measurement of independent variables, and the complex mapping relation between numerous independent variables and disease metrics.

Based on the above requirement analysis and comparison of existing forecasting technologies, we proposed the PHTFNet-RPM to build the forecasting model according to the temporal fusion transformer (Lim et al., 2021; Nazir et al., 2023) and the Bayesian transformers (Sankararaman et al., 2022; Xiao et al., 2024). Comparing with the previous works, e.g., the CNN-LSTM for forecasting tobacco diseases (Chen et al., 2025) and the LSTM-Attention for forecasting the pest incidence in tea gardens (Chen et al., 2024), the uncertainty estimate based on Bayesian deep learning (Sankararaman et al., 2022) is integrated in our model for robust and reliable prediction. Especially, a Random Period Mask (RPM) mechanism is designed in the model learning so that our model better processes the various absences of past observed disease incidence and disease index. It improves the robustness and flexible forecasting capability of model because it is possibly that there are partial disease survey records and sometimes even no records during the manual collection in the practical applications.

**Figure 3:**
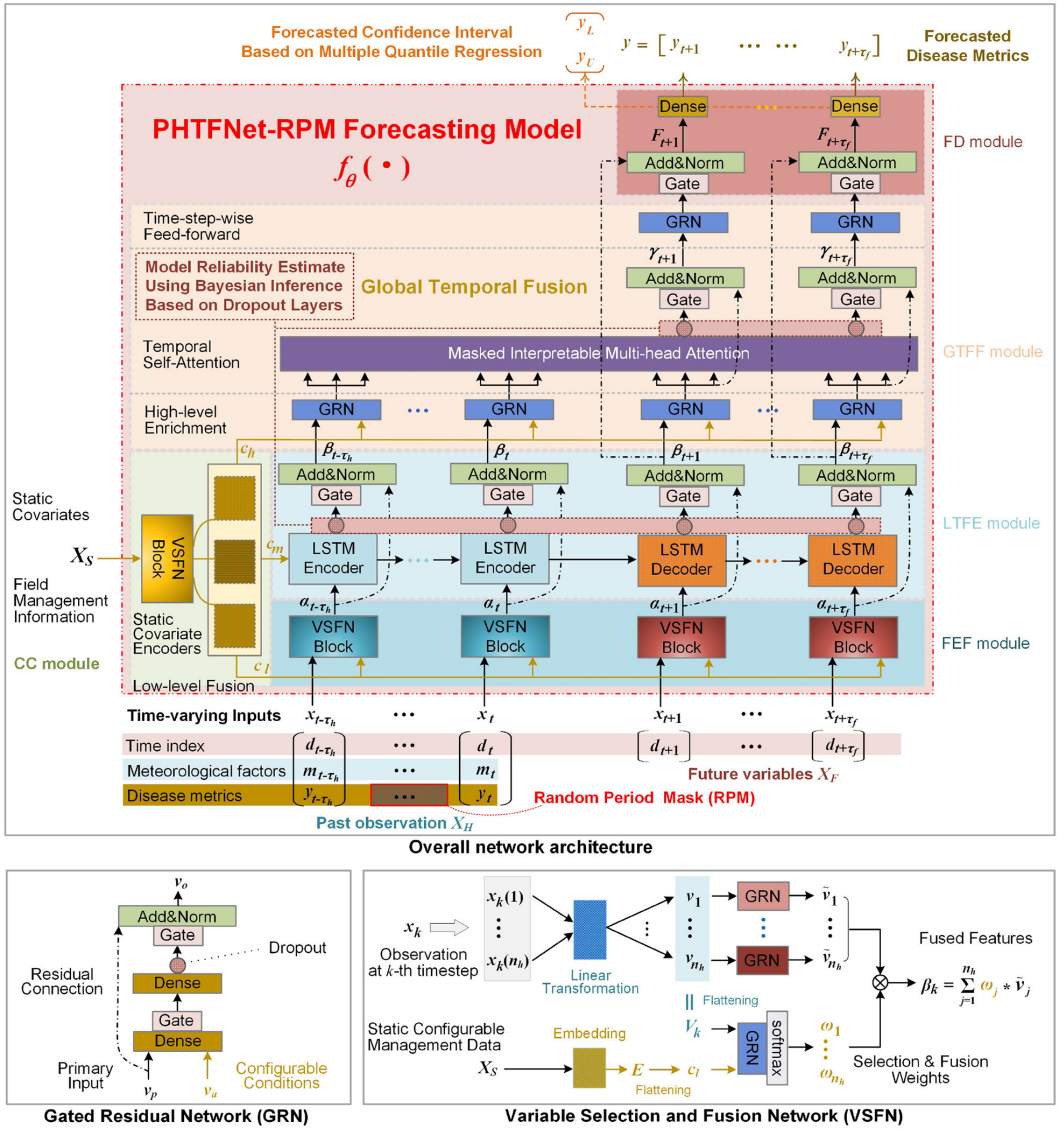
Overall network architecture and built-in subnetworks of GRN and VSFN. CC, condition configuration; FEF, feature extraction & fusion; LTFE, local temporal feature exploration; GTFF, global temporal feature fusion; FD, forecasting decision.

The proposed PHTFNet-RPM in Figure 3 mainly contains five modules: Condition Configuration (CC), Random Period Mask (RPM), Feature Extraction and Fusion (FEF), Local Temporal Feature Exploration (LTFE), Global Temporal Feature Fusion (GTFF), and Forecasting Decision (FD). All the modules are sequentially connected and integrated to construct the forecasting model *f*_θ_(·), where θ indicates the learnable parameters in the whole neural network. The connected arrows between these modules describe the information flows from model input to prediction results. In the following parts, the common block in multiple modules, i.e. Gated Residual Network (GRN), is firstly described. Then, according to the information flow direction from model input to model output, each module is then introduced in details.

#### 3.2.2 Complexity-adaptive GRN

GRN is an embedded learnable neural network block with flexible and powerful nonlinear expressive capabilities (Lim et al., 2021). It is deployed in multiple modules to improve the model's nonlinear representational capacity as shown by the GRN in Figure 3. Usually, it is fed two input vectors, i.e., primary vector *v*_*p*_ and auxiliary input *v*_*a*_. As shown by the VSFN in Figure 3, *v*_*p*_ and *v*_*a*_ are firstly concatenated to perform a simple yet effective nonlinear transformation with an Exponential Linear Unit (Clevert et al., 2015). Then, a non-linear Gated Linear Unit is followed to enhance the feature selection capability for critical features (Clevert et al., 2015). In addition, a Dropout layer is added to avoid overfitting and improve its generalization ability (Srivastava et al., 2014). If the above three transformations are together denoted as a non-linear function φ__*EGD*__(·), when the residual connection mechanism is applied, then the output *v*_*o*_ of GRN block is described as:


$$v_{o}=\text{LN}(v_{p}+\varphi_{EGD}([v_{p},v_{a}])),$$ (3)

where *LN*(·) indicates a LayerNorm operation. To sum up, the nonlinear expressive capability of GRN block is handled by φ__*EGD*__(·). Its adaptability to complexity is reflected by the residual connection and the gating mechanism in φ__*EGD*__(·). For example, when the output of φ__*EGD*__(·) approaches a zero vector, the output *v*_*o*_ is approximated to the primary input *v*_*p*_. Depending on the learned parameters optimized by the data-driven training, the GRN can play different roles, ranging from simple linear transformations to complex nonlinear mappings.

#### 3.2.3 Hybrid-structured model input

##### 3.2.3.1 Time-series input with RPM

In practical applications, for past observation, time-series weather data is easily accessible. However, historically continuous records of disease incidence and disease index are notoriously difficult to acquire, which is caused by two reasons: (1) The manual investigation of disease statuses is complex and may be subject to delays or missing data due to investigator negligence. (2) The labor cost of the investigation is high. In some areas, the disease incidence and disease index may only be surveyed once every 5 or 10 days. As a result, past observational data is not available on a daily basis. Especially, in some planting areas, it is required to forecast disease risks only using the weather data and management information under the condition of no past disease data. To enable the model to make predictions in such scenarios, the training data must simulate the scenarios so that the model can learn the corresponding inference capability. To this end, the RPM is designed to handle time-series input as shown by the overall architecture in Figure 3. The process can be described as:


$$\begin{array}{l} X_{H}=X_{H}⊙M_{z}+M_{b}, \end{array}$$ (4)

where $X_{H},M_{z},M_{b}\in {\mathbb{R}}^{n_{h}\times \left(\tau_{h}+1\right)}$. If denote *A* as the mask area for disease incidences and disease indices in *X*_*H*_, then *M*_*z*_(*i, j*) = 0 for the index coordinate (*i, j*)∈*A*, otherwise *M*_*z*_(*i, j*) = 1. Similarly, *M*_*b*_(*i, j*) = −1 for (*i, j*)∈*A*, otherwise *M*_*b*_(*i, j*) = 0. In this way, for *X*_*H*_, its elements in the mask area *A* are set as −1. For the mask area *A*, it can be generated using two random integers, the start day and mask period days.

##### 3.2.3.2 CC module for configurable static input

When the model conducts forecast, the static discrete variables describing field management information, such as location, disease category and others shown in Table 1, are required and combined with time-varying variables. These configurable inputs represent different forecast requirements and planting conditions. They affect feature extraction of model in different levels. To this end, the CC module adopts three learnable feature embedding blocks to encode the discrete input *X*_*S*_ to obtain three feature vectors *c*_*l*_, *c*_*m*_ and *c*_*h*_. They are respectively fused into low-level, medium-level and high-level feature extraction in the total networks. For example, *c*_*l*_ is integrated into the FEF module to combine time-varying variables to extract low-level features. *c*_*m*_ is used to initialize the cell state and hidden state of the first LSTM Encoder for better exploiting local temporal features in medium level. *c*_*h*_ is fed to the GTFF to help capture global temporal dependent features related to the configurable input in high-level semantics. Although the configurable static input containing field management information is helpful to performance improvement, there is an additional cost associated with collecting field management information. Moreover, the static input is tailored to specific crops and diseases, and its configuration varies depending on the application. For instance, when forecasting diseases for other crops, it is necessary to reselect the relevant field management variables and determine the appropriate embedding coding through experimentation.

#### 3.2.4 Timestep-wise feature extraction and fusion using VSFN

The FEF module is primarily responsible for selecting and fusing multiple variables, and then extracting low-level features for each time step. This is mainly implemented by the VSFN in Figure 3. The historical observation *X*_*H*_ shares a VSFN, and the future data *X*_*F*_ shares the other VSFN, as shown by the overall architecture in Figure 3. Next, we take the historical observation *X*_*H*_ as an example to describe the function of VSFN. Denote $x_{k}\in {\mathbb{R}}^{n_{h}}$ indicate multi-variable observation data at the *k*-th time step, where *x*_*k*_∈*X*_*H*_, *t*−τ_*h*_ ≤ *k* ≤ *t*, *t* is the forecasting time. Firstly, by a learnable linear transformation, *x*_*k*_ is transformed as a matrix, which contains *n*_*h*_ vectors [*v*_1_, ⋯ , *v*_*n*_*h*__]. Namely, the observed scalar value of each variable *x*_*k*_(*j*), *j* = 1, ⋯ , *n*_*h*_, is lifted as a *n*_*e*_-dimension vector *v*_*j*_. Then, the GRN follow it to obtain ṽ_*j*_, which improves the nonlinear representational capacity. At the same time, the feature data [*v*_1_, ⋯ , *v*_*n*_*h*__] for time-varying variables and the feature data *c*_*l*_ for static variables are combined to calculate a *n*_*h*_-dimension weight vector [ω_1_, ⋯ , ω_*n*_*h*__] for selecting and fusing features of time-varying variables. For the input (*x*_*k*_, *c*_*l*_) at the *k*-th time step, the output of VSFN is finally computed as:


$$\begin{array}{l} \alpha_{k}=\overset{n_{h}}{\underset{j=1}{\sum }}\;\omega_{j}\;{ṽ}_{j}. \end{array}$$ (5)

It will be fed to the *k*-th LSTM encoder in the followed LTFE module.

#### 3.2.5 LTFE module for across-timestep local temporal feature exploitation

The LTFE module aims to exploit useful across-timestep feature patterns from temporally adjacent low-level features [α_*t*−_τ__*h*__, ⋯ , α_*t*_, ⋯ , α_*t*+_τ__*h*__]. The sequence-to-sequence LSTM encoding and decoding architecture (Park et al., 2018) and the residual mechanism are adopted to enhance the low-level α_*k*_ from each time step in the FEF module. Let φ__*LSTM*__(·) denote the LSTM encoding and decoding function, then the output β_*k*_ at the *k*-th time step can be described as:


$$\begin{array}{l} \beta_{k}=\text{LN}(\alpha_{k}+\varphi_{LSTM}(c_{m},[\alpha_{t-\tau_{h}},\cdots \;,\alpha_{k}])), \end{array}$$ (6)

where *LN*(·) indicates the LayerNorm operation. The encoded feature vector *c*_*m*_ of static variables is used to initialize the the cell state and hidden state of the first LSTM Encoder. Through the process of state backpropagation, the input gates and forget gates in the subsequent LSTM units are capable of retaining disease-relevant information while discarding information unrelated to the disease. Relying on the flow of information between memory cells within adjacent LSTM units, φ__*LSTM*__(·) can explore the useful feature patterns from the past observation [α_*t*−_τ__*h*__, ⋯ , α_*k*_] before the *k*-the time step, e.g. temporally-local weather variations and modifications of disease statuses.

#### 3.2.6 GTFF module for attention-focused global temporal feature fusion

The GTFF module is designed to learn the temporally-global helpful feature patterns from the past observation, e.g. the past rainfalls, the average temperature, and the variation trends of weather factors etc. Firstly, GRN is adopted to fuse the input features *B* = [β_*t*−_τ__*h*__, ⋯ , β_*t*+_τ__*f*__] and the static encoded vector *c*_*h*_ to obtain $\tilde{B}=\left[{\tilde{\beta }}_{t-\tau_{h}},\cdots \;,{\tilde{\beta }}_{t+\tau_{f}}\right]$. Then, an interpretable multi-head attention block (Lim et al., 2021) is adopted to extract temporally-global attention-focused features Γ as:


$$\begin{array}{l} \Gamma =\frac{1}{n_{H}}\overset{n_{H}}{\underset{h=1}{\sum }}\Phi_{Att}\left(\tilde{B}\;W_{K}^{h},\;\tilde{B}\;W_{Q}^{h},\;\tilde{B}\;W_{V}\right), \end{array}$$ (7)

where $\Gamma \in {\mathbb{R}}^{n_{e}\times \tau_{f}}$. Φ_*Att*_(·) indicates a multi-head self-attention operation (Vaswani et al., 2017). *n*_*H*_ is the number of heads, $W_{K}^{h}$ and $W_{Q}^{h}$ are respectively the learnable weights for the keys and queries in the *h*-th head. *W*_*V*_ indicates a across-head learnable weight (Lim et al., 2021). Denote Γ(*t*+1), ⋯ , Γ(*t*+τ_*f*_) are the temporally-global attention-focused features at the forecasting timesteps. They are combined with ${\tilde{\beta }}_{t+1},\cdots \;,{\tilde{\beta }}_{t+\tau_{f}}$ to generate the output of GTFF module according to the following mean:


$$\begin{array}{l} \gamma_{k}=LN\left({\tilde{\beta }}_{t+1}+\varphi_{GLU}\left(\Gamma \left(k\right)\right)\right), \end{array}$$ (8)

where *k* = *t*+1, ⋯ , *t*+τ_*f*_, φ_*GLU*_(·) indicates a standard GLU operation, *LN* is a LayerNorm operation. For the *k*-th forecasting time step, this mean effectively fuses the temporally-global attention-focused feature Γ(*k*) and the previous temporally-local feature ${\tilde{\beta }}_{k}$. Especially, in model learning, the decoder masking (Li et al., 2019) is adopted to enable that each time step in the decoding stage only pay attention to the information preceding it.

#### 3.2.7 FD module for multi-horizon and multi-target prediction

The FD module combines the sequence-to-sequence decoded features and the temporally-global self-attention focused features to conduct multi-horizon and multi-target prediction. For the future *k*-th time step, *k*∈{*t*+1, ⋯ , *t*+τ_*f*_}, the final prediction feature vector *F*_*k*_ and the predicted values of target variables are respectively calculated as:


$$\begin{array}{l} F_{k}=\;LN\left(\;\beta_{k}+\varphi_{GG}\left(\gamma_{k}\right)\;\right), \end{array}$$ (9)

$$\begin{array}{l} y_{k}=W_{k}F_{k}+b_{k}. \end{array}$$ (10)

Here, φ_*GG*_(·) denotes the GRN block and the gating layer, and *LN*(·) indicate the normalization layer in the network as shown by the overall architecture in Figure 3. The predicted disease metric $y_{k}\in {\mathbb{R}}^{2}$ is a 2-dimension vector containing disease incidence and disease index. *W*_*k*_ and *b*_*k*_ are learnable parameters. Especially, when the multi-quantile regression loss (Cai, 2002) is adopted to train the model for evaluating the confidence interval of predicted disease metric, multiple outputs *y*_*k, q*_ for different quantiles *q*∈*Q* = {0.1, 0.5, 0.9} are required, which will be introduced in Section 3.3.2.1. Correspondingly, multiple sets of parameters {*W*_*k, q*_, *b*_*k, q*_} will be learned. In this way, multi-horizon and multi-target predictions at different quantiles, *y*_*k, q*_ = *W*_*k, q*_*F*_*k, q*_+*b*_*k, q*_, *k* = *t*+1, ⋯ , *t*+τ_*f*_, are simultaneously obtained.

In summary, the DDN-based *f*_θ_(·) consists of multiple modules and each module contains a large number of learnable parameters. The symbol θ represents all learnable parameters in the networks, which can be optimized by a data-driven learning mean.

### 3.3 Model learning and inference

In the real-world systems, besides the prediction performance, practitioners are also concerned about confidence and robustness of predictions because unreliable predictions may result in incorrect management decisions causing economic losses. The unreliable predictions primarily stem from data uncertainty and model uncertainty. To estimate the reliability of prediction, considering the computation cost, the predicted quantile (credible) interval is used to present the uncertainty of a predicted result. The interval can be easily implemented by augmenting the network outputs at multiple quantiles and supervising the learning process using the quantile regression loss (Koenker, 1994; Cai, 2002; Lim et al., 2021). The uncertainty (reliability) estimate of model is mainly concerned by researchers for consistently improving the forecasting model. The approximate variational inference based on Dropout in Bayesian deep learning (Kendall and Gal, 2017; Sankararaman et al., 2022) is adopted to evaluate it in model learning and inference. In the following parts, they will be discussed in detail.

#### 3.3.1 Model learning

It is a regression task to predict the disease incidence and disease index. To optimize the parameter θ in the model *f*_θ_(·), a loss function supporting regression task is required to implement data-driven training process using the dataset *D* = {(*X*_*i*_, *y*_*i*_)|*i* = 1, ⋯ , *n*}. The quantile regression loss allows the model to simultaneously output predictions at different quantiles and thus generate probabilistic forecasting in low computation cost. Formally, by jointly minimizing the quantile losses, the optimal parameters θ^*^ is solved by training the parameterized network using *D*:


$$\begin{array}{l} \theta^{*}=\underset{\theta }{\arg\min}\;\ell \left(D,f_{\theta }\left(\cdot \right)\right), \end{array}$$ (11)

$$\begin{array}{l} \ell \left(D,f_{\theta }\left(\cdot \right)\right)= \end{array}$$ (12)

$$\begin{array}{l} \frac{1}{|Q||D|\tau_{f}\;n_{m}}\underset{q\in Q}{\sum }\;\underset{\left(X_{i},y_{i}\right)\in D}{\sum }\;\overset{\tau_{f}}{\underset{k=1}{\sum }}\;\overset{n_{m}}{\underset{j=1}{\sum }}L_{q}\left(y_{i}^{\prime }\left(q,k,j\right),y_{i}\left(q,k,j\right)\right), \end{array}$$

$$\begin{array}{l} L_{q}\left(x_{1},x_{2}\right)=\max\left(q\left(x_{2}-x_{1}\right),\left(1-q\right)\left(x_{1}-x_{2}\right)\right), \end{array}$$ (13)

where *Q* = {0.1, 0.5, 0.9}, ℓ(·, ·) indicates the total loss, and *L*_*q*_(·, ·) is the quantile regression loss function (Cai, 2002). $y_{i}^{\prime }=f_{\theta }\left(X_{i}\right)$ is the predict result for *X*_*i*_. $y_{i}^{\prime }\left(q,k,j\right)$ denotes the *q*-quantile prediction for the *j*-th target variable on the future *t*+*k* day. *y*_*i*_(*q, k, j*) is its corresponding ground-truth.

#### 3.3.2 Model inference and performance evaluation

##### 3.3.2.1 Probatilistic predictions

Let $f_{\theta^{*}}\left(\cdot \right)$ indicates the learned network model disabling the Dropout layers, for a test sample *X*, the prediction $y^{\prime }=f_{\theta^{*}}\left(X\right)$ is a three-dimensional matrix $y^{\prime }\in {\mathbb{R}}^{n_{q}\times \tau_{f}\times n_{m}}$. It indicates the predicted values of *n*_*m*_ target variables in the future τ_*f*_ days for *n*_*q*_ quantiles. For notational convenience, by omitting *k* and *j*, we directly denote *y*′(*q, k, j*) as $y_{q}^{\prime }$, which indicates the *q*-quantile prediction value of the *j*-th target variable in the future *k*-th day. Namely, the values $y_{0.1}^{\prime }$, $y_{0.5}^{\prime }$ and $y_{0.9}^{\prime }$ individually indicate the predicted values at the quantiles of 0.1, 0.5 and 0.9. The value $y_{0.5}^{\prime }$ indicates the point prediction, which is approximately equivalent to the prediction of model driven by the Mean Absolute Error (MAE) loss (Cai, 2002). The interval $\left[y_{0.1}^{\prime },y_{0.9}^{\prime }\right]$ can be thought as the confidence interval [*y*_*L*_, *y*_*U*_] as shown by the overall architecture in Figure 3. If the interval is large, the uncertainty of the point prediction $y_{0.5}^{\prime }$ is high and the prediction result is with low reliability.

##### 3.3.2.2 Performance metrics

For forecasting the diseases, it is important to correctly predict the increasing or decreasing trend of disease progression and point-wise anomalies are less consequential. On the other hand, the low outlier of prediction is also significant because the negative impact of underestimating the disease progression is far greater than that of overestimating it. Based on the above analysis, the robust and outlier-insensitive Mean Absolute Error (MAE) is adopted to evaluate the model performance on *D*_*test*_ using the *q* = 0.5 quantile output (point prediction):


$$MAE=\frac{1}{n_{T}\;\tau_{f}\;n_{m}}\sum_{i=1}^{n_{T}}\sum_{k=1}^{\tau_{f}}\sum_{j=1}^{n_{m}}\left|y_{i}^{'}\left(q,k,j\right)-y_{i}\left(q,k,j\right))\right|.\;$$ (14)

For the performance on the low outlier of prediction, the *q*-risk at *q* = 0.1 is adopted:


$$\begin{array}{l} q\text{-}Risk=\frac{2\overset{n_{T}}{\underset{i=1}{\sum }}L_{q}\left(y_{i}\left(q,k,j\right),y_{i}^{\prime }\left(q,k,j\right)\right)}{\overset{n_{T}}{\underset{i=1}{\sum }}|y_{i}\left(q,k,j\right)|}. \end{array}$$ (15)

Here, *n*_*T*_ is the number of samples in *D*_*test*_. $y_{i}^{\prime }\left(q,k,j\right)$, *y*_*i*_(*q, k, j*)) and *L*_*q*_(·, ·) are same to Equations 12, 13. When fixing *k* or *j*, the MAE and *q*-Risk on a certain day or for a certain disease metric can also be evaluated. For notational convenience, the *q*-Risk at *q* = 0.1 is denoted as *P*10 in the following experiment results.

##### 3.3.2.3 Reliability of model

The Monte Carlo Dropout-based Bayesian inference (Xiao et al., 2024) is used to estimate the uncertainty of model by the Dropout layers as shown by the overall architecture in Figure 3. Based on the solved model $f_{\theta^{*}}\left(\cdot \right)$, the Dropout operations are repeatedly conducted for *n*_*r*_ times of sampling parameters from the distribution of model parameter θ, then the *n*_*r*_ models, denoted as ${\tilde{f}}_{\theta_{1}^{*}}\left(\cdot \right),\cdots \;,{\tilde{f}}_{\theta_{n_{r}}^{*}}\left(\cdot \right)$, are obtained. Let *X*_1_, ⋯ , *X*_Ñ_ is a subset from *D*_*test*_, the model uncertainty *Var*(*y*) for the learned model $y=f_{\theta^{*}}\left(X\right)$ is defined:


$$\begin{array}{l} Var\left(y\right)=\frac{1}{Ñ}\overset{Ñ}{\underset{i=1}{\sum }}\left(\frac{1}{n_{r}-1}\overset{n_{r}}{\underset{j=1}{\sum }}{\left({\tilde{f}}_{\theta_{k}^{*}}\left(X_{i}\right)-\frac{1}{n_{r}}\overset{n_{r}}{\underset{j=1}{\sum }}{\tilde{f}}_{\theta_{k}^{*}}\left(X_{i}\right)\right)}^{2}\right). \end{array}$$ (16)

The previous researches (Kendall and Gal, 2017) proved that the uncertainty is medium when *Var*(*y*) falls in [0.01, 0.1]. This shows that the model is reliable for such samples because the tested samples and training samples have the similar distribution. When *Var*(*y*) is higher than 0.1, it means that the input samples are unusual and the model is unreliable. It is important to estimate the model uncertainty and reliability when the model is new deployed or when the model is updated by new training data.

## 4 Experiment analysis

In this part, the experiment settings are firstly described in detail. Then, comparative experiments between different methods are conducted to verify the proposed forecasting method by evaluating the performances of MAE and P10 metrics. Further, we conduct an ablation study to analyze the modules in the model *f*_θ_(·) and the independent variables in model input so that their respective contributions to forecasting accuracy are demonstrated.

### 4.1 Experiment settings

#### 4.1.1 Data settings

During data preparation, for two discrete variables of survey year and sampling date (time index), considering their continuous variation, we converted them to real-valued representations by the min-max normalization. For the other discrete variables in Table 1, we took them as categorical variables. For the continuous variables except for target variables, the *z*-score normalization was applied across all time-series entities. After the above data preprocessing, the collected dataset is randomly divided into three groups: training set *D*_*train*_ for model learning, validation set *D*_*val*_ for hyperparameter tuning and test set *D*_*test*_ for performance evaluation according to the ratio of (7:1:2).

#### 4.1.2 Model configuration and training settings

To better match the problem's complexity, several key hyperparameters of model *f*_θ_(·) must be determined, including the dimension of low-level feature encoding, the encoder/decoder lengths in the sequence-to-sequence architecture, the hidden state size, and the Dropout rate. Moreover, in the process of training *f*_θ_(·), to achieve the optimal $f_{\theta^{*}}\left(\cdot \right)$, we need to set the other hyperparameters, e.g. the Optimizer, the learning rate, the batch size etc. This work adopted the random search method to set the hyperparameters' values by evaluating performances on *D*_*val*_. The optimal settings of hyperparameters are shown in Table 2. For the other compared methods, the related hyperparemeters are set according to the corresponding literatures. Pytorch forecasting package (see text footnote 1) is adopted to design the model and conduct experimental analysis. The models are trained and evaluated on the GPU of Nvidia RTX 4090.

**Table 2:** Configuration of model hyperparameters and settings of hyperparameters in training process.

**Hyperparameter**	**Optimal value**	**Description**
*n*_*e*_- state size	60	Hidden state size in networks.
τ_*h*_- Max history days	10	Sequence-to-sequence encoding length.
τ_*f*_- Max forecast days	3	Sequence-to-sequence decoding length.
*p*-Dropout rate	0.3	In the Dropout layers.
*n*_*H*_-Number of heads	4	Self-attention in Equation 7.
Batch size	64	Number of samples input in an Epoch.
Learning rate	0.001	A parameter of optimizer.
Max gradient norm	0.01	Avoid exploding gradients in training.
Optimizer	AdamOptimizer	Algorithm of updating parameters.

The top six rows are model hyperparameters. The others are hyperparameters for training.

### 4.2 Comparative experiments

To validate the proposed forecast method, we compare ours with the previous popular approaches for forecasting crop disease risks. The compared models consist of two categories: the canonical models and the NN-based models. For the canonical models, we mainly consider two baselines: the Linear Regression (LR) and the kernelized non-linear Support Vector Regression (SVR). LR was even used to predict the risks of rust Cercospora and tobacco Caterpillar (Patel et al., 2020). SVR is popular in forecasting disease risks (Fenu and Malloci, 2021), e.g. forecasting wildfire disease in Cai et al. (2019). For the NN-based models, Multi-Layer Perceptron (MLP) (Tammina et al., 2024), CNN-LSTM (Wang et al., 2024; Chen et al., 2025), and LSTM-Attention (Chen et al., 2024) are compared. More details are described as follows:

- LR: all available information at each time-step and static management information were concatenated as one total vector as the input of the linear regression with *L*_2_ regularization. Multi-quantile regression loss was used to drive the model learning. For each horizon/quantile output, an individual set of parameters were learned (Patel et al., 2020).
- SVR: the above concatenated vector serves as the SVR's input. The Radial Basis Function (RBF) kernel was employed to enhance the nonlinear modeling capability (Fenu and Malloci, 2021).
- MLP: a two-layered neural network with the ReLU units was constructed to predict multi-quantile outputs in the future time-steps (Tammina et al., 2024). Its input was same to the above setting in LR and SVR.
- CNN-LSTM: according to the network architectures for forecasting cucumber downy mildew disease (Wang et al., 2024) and tobacco diseases (Chen et al., 2025), a CNN is used to excavate the features between adjacent time-steps for single variable and the integrated features across multiple variables. Then a LSTM layer is stacked to further develop the useful features across time-steps.
- LSTM-attention: according to the time-series forecasting model for insect pests in tea gardens (Chen et al., 2024), attention blocks inspired by the self-attention mechanism in the Transformer network are embedded between LSTM encoding and encoding blocks to distill the long-distance temporal dependences, which is helpful to capture the useful features on specific time-steps.

In this experiment, we simulated the real-world application scenarios where recent 5-day past observations of disease metrics were masked by RPM in model learning and inference. To achieve the robust evaluation of performances, splitting *D*_*train*_, *D*_*val*_ and *D*_*test*_ from the original *D* were repeatedly conducted multiple times, five times of evaluations on them were averaged to report the final MAE and P10. For fair comparison, for other approaches, the multi-quantile regression loss was also used to train them. The optimal hyper-parameters for different models are experimentally determined on *D*_*val*_.

Table 3 shows the forecast performances (MAE and P10) of disease incidence and disease index for two diseases. Obviously, our model significantly outperforms the other models. For instance, in forecasting the disease incidence of black shank, for the comprehensive performance metrics of Avg. MAE and Avg. P10, our model achieves a 16.43% reduction on MAE and a 14.97% reduction on P10 *q*-risk compared to the baseline LR model. Compared to the suboptimal model (LSTM-Attention), our model respectively reduces MAE by 5.93% and P10 by 4.69%. In forecasting the disease index of black shank, compared to the LR and LSTM-Attention models, 15.94% reduction and 4.79% reduction on Avg. MAE are individually achieved, and 14.56% reduction and 3.35% reduction on Avg. P10 are obtained respectively. For the black root rot, the similar performance improvements are achieved. In summary, all contrastive results demonstrate that our model achieves significantly higher accuracy in predicting both disease incidence and disease index for the upcoming 3-day period.

**Table 3:** Prediction performances (MAEs and P10s) of disease incidence (%) and disease index (%) for different forecast methods on two diseases.

**Diseases**	**Methods**	**1-day ahead MAE**	**2-day ahead MAE**	**3-day ahead MAE**	**Avg. MAE**	**1-day ahead P10**	**2-day ahead P10**	**3-day ahead P10**	**Avg. P10**
**Prediction performances of disease incidence**									
Black shank	LR	0.1056	0.1114	0.1243	0.1138	0.0423	0.0487	0.0594	0.0501
	SVR	0.0997	0.1041	0.1166	0.1068	0.0401	0.0462	0.0564	0.0476
	MLP	0.1005	0.1058	0.1193	0.1085	0.0401	0.0466	0.0569	0.0479
	CNN-LSTM	0.0954	0.0998	0.1118	0.1023	0.0378	0.0442	0.0537	0.0452
	LSTM-Attention	0.0939	0.0979	0.1116	0.1011	0.0374	0.0440	0.0525	0.0447
	**PHTFNet-RPM**	**0.0913**	**0.0917**	**0.1022**	**0.0951**	**0.0361**	**0.0420**	**0.0496**	**0.0426**
Black root rot	LR	0.0873	0.0965	0.1047	0.0962	0.0374	0.0431	0.0509	0.0438
	SVR	0.0821	0.0897	0.0978	0.0899	0.0353	0.0407	0.0482	0.0414
	MLP	0.0827	0.0913	0.1001	0.0914	0.0353	0.0411	0.0486	0.0416
	CNN-LSTM	0.0790	0.0867	0.0943	0.0867	0.0335	0.0392	0.0461	0.0396
									
	LSTM-Attention	0.0779	0.0851	0.0943	0.0858	0.0332	0.0390	0.0452	0.0391
	**PHTFNet-RPM**	**0.0761**	**0.0803**	**0.0870**	**0.0811**	**0.0322**	**0.0375**	**0.0430**	**0.0375**
**Prediction performances of disease index**									
Black shank	LR	0.0764	0.0813	0.0908	0.0828	0.0316	0.0362	0.0435	0.0371
	SVR	0.0718	0.0756	0.0848	0.0774	0.0298	0.0342	0.0412	0.0351
	MLP	0.0722	0.0767	0.0867	0.0785	0.0298	0.0344	0.0414	0.0352
	CNN-LSTM	0.0688	0.0727	0.0815	0.0743	0.0282	0.0328	0.0392	0.0334
	LSTM-Attention	0.0675	0.0709	0.0811	0.0731	0.0278	0.0325	0.0382	0.0328
									
	**PHTFNet-RPM**	**0.0663**	**0.0673**	**0.0751**	**0.0696**	**0.0271**	**0.0314**	**0.0365**	**0.0317**
Black root rot	LR	0.0373	0.0392	0.0445	0.0403	0.0157	0.0174	0.0216	0.0182
	SVR	0.0348	0.0361	0.0412	0.0374	0.0147	0.0163	0.0203	0.0171
	MLP	0.0353	0.0371	0.0426	0.0383	0.0148	0.0166	0.0206	0.0173
	CNN-LSTM	0.0337	0.0352	0.0405	0.0363	0.0141	0.0158	0.0195	0.0165
	LSTM-Attention	0.0333	0.0346	0.0401	0.0360	0.0139	0.0159	0.0192	0.0163
									
	**PHTFNet-RPM**	**0.0328**	**0.0330**	**0.0374**	**0.0344**	**0.0136**	**0.0153**	**0.0184**	**0.0158**

The smaller the value, the better. The Avg. MAE and Avg. P10 mean that the performance is evaluated across all forecasting time-steps. The MAEs are small because the disease incidences are low in most years. Bold values indicate the best value across different models.

By further contrastive analysis on the MAE and P10, we systematically examine the underlying factors contributing to the divergent prediction performances among models in Table 3. Firstly, the LR model is the simple linear model and it achieves the lowest prediction performance because the relation between the growing conditions of tobacco plants and the disease severity is intricately non-linear. It is difficult for a linear model to accurately depict such relation. Subsequently, the second-tier models comprise SVR and MLP, which improve the performance by about 5% compared to the LR. The improvement mainly comes from their non-linear expressive capability. However, the improvements are limited because the variations across time-steps cannot be effectively modeled in them. The remaining comparable models using complex hybrid architectures, i.e. CNN-LSTM, LSTM-Attention and ours, fall in the time-series forecast models. Compared to the CNN-LSTM and LSTM-Attention, our performance improvement mainly comes from multiple factors: (1) Low-level adaptive variable selection and fusion; (2) Middle and high-level feature representations including short and long-range temporal dependency modeling; (3) multi-level fusion of static and time-series variables; (4) Random Period Mask in training for better addressing data missing of disease records, which will be further discussed in the last Section.

To delve deeper, we compare ours with CNN-LSTM and LSTM-Attention to analyze the model capability and robustness across prediction horizons, disease categories, different forecast targets by multi-perspective investigation of their results in Table 3. By comparing MAEs or P10s along the 1-, 2-, and 3-day ahead predictions for each model, the accuracy degradation for horizon-dependent prediction is available for all the models. But the degradation for ours is much slower than the other two models. For example, for the MAE of predicted disease incidence for black shank, by comparing 3- and 1-day ahead predictions, the degradations of 11.9% for ours and 18.8% for LSTM-Attention are individually obtained. The lower MAE or P10 *q*-risk together with the slower degradation along forecast horizon proves the superior modeling capability of $f_{\theta^{*}}\left(\cdot \right)$. Namely, ours can extract effective time-step-wise features from daily weather factors and planting management information, better model the temporal dependent relations between time steps, including short-range and long-range ones.

Finally, we discuss the model robustness and reliability by quantizing the model uncertainty and analyzing the *q*-risk P10. To estimate the model uncertainty, 20% of samples in *D*_*test*_ are randomly selected and *Var*(*y*) is computed according to Equation 16. According to the previous researches (Kendall and Gal, 2017), the evaluated *Var*(*y*) = 0.028 shows the uncertainty mainly comes from the aleatoric uncertainty (data noises), rather than the epistemic uncertainty (underperforming model). This validates the reliability of our model. In addition, for a test sample *X*, when evaluating its prediction target *y*′ at a time step, a confidence interval $\left[y_{0.1}^{\prime },y_{0.9}^{\prime }\right]$ can be obtained at the same time, which means $P\left(y_{0.1}^{\prime }\leq y\leq y_{0.9}^{\prime }\right)=0.8$ for the ground truth *y*. Both the predicted confidence interval $\left[y_{0.1}^{\prime },y_{0.9}^{\prime }\right]$ and the relative position of *y*′ in the interval exhibit the reliability of predicted results. The narrower the predicted interval and the closer the forecast value is to the midpoint of the interval, the more reliable the predicted result. Conversely, wider intervals and values farther from the center indicate lower reliability. This allows agronomists to make informed management decisions based on this predictive data. Especially, by investigating the results of *q*-risk P10 (*q* = 0.1) in Table 3, our model achieves the lowest *q*-risk compared to the other models. This shows that our model can effectively control the lower tail risk. In summary, the above experimental results verify that our model is reliable when deploying it to forecast the tobacco root diseases.

### 4.3 Model analysis and discussion

In this part, we firstly conduct visualization analysis, for instance, executing rolling prediction and plotting the long-range predictive results for visual inspection of prediction efficacy and investigating whether accurately predict the increasing or decreasing trends of disease progression. Then, ablation experiments are designed to analyze the roles of key modules in the network model *f*_θ_(·) or contributions of different observed variables. Finally, temporal feature patterns are inspected to discuss what historical events or information contribute more to accurately forecasting different diseases.

#### 4.3.1 Visualization of predictive disease progression trend

In forecasting tobacco diseases, it is very difficult to accurately predict daily disease incidence or disease index in the future days due to the complicated disease progression caused by the varying growth conditions and manual managements. From the viewpoint of disease management, monitoring disease severity progression trends (increase/decrease) demonstrates greater clinical significance compared to daily disease index tracking. Therefore, we design an experiment to investigate the model's capability in predicting the progression trend of diseases.

In this experiment, for each disease, we randomly select a time-series entity in a monitoring location, which contains daily weather data, disease records (disease incidence and disease index) and management information in the total disease progression period. Then, the model $f_{\theta^{*}}\left(\cdot \right)$ is used to conduct rolling prediction and generate the total predictive progression trend. The predictive process proceeds iteratively as follows: Observations from the start date (e.g. May 10) to May 19 are used to predict the target value on May 20; subsequently, observations from May 11 to May 20 predict the target value on May 21. This sliding-window forecasting scheme is executed iteratively until all target values are generated, thereby constructing a complete predictive trend. The settings of model learning and inference are same to the previous comparative experiments.

**Figure 4:**
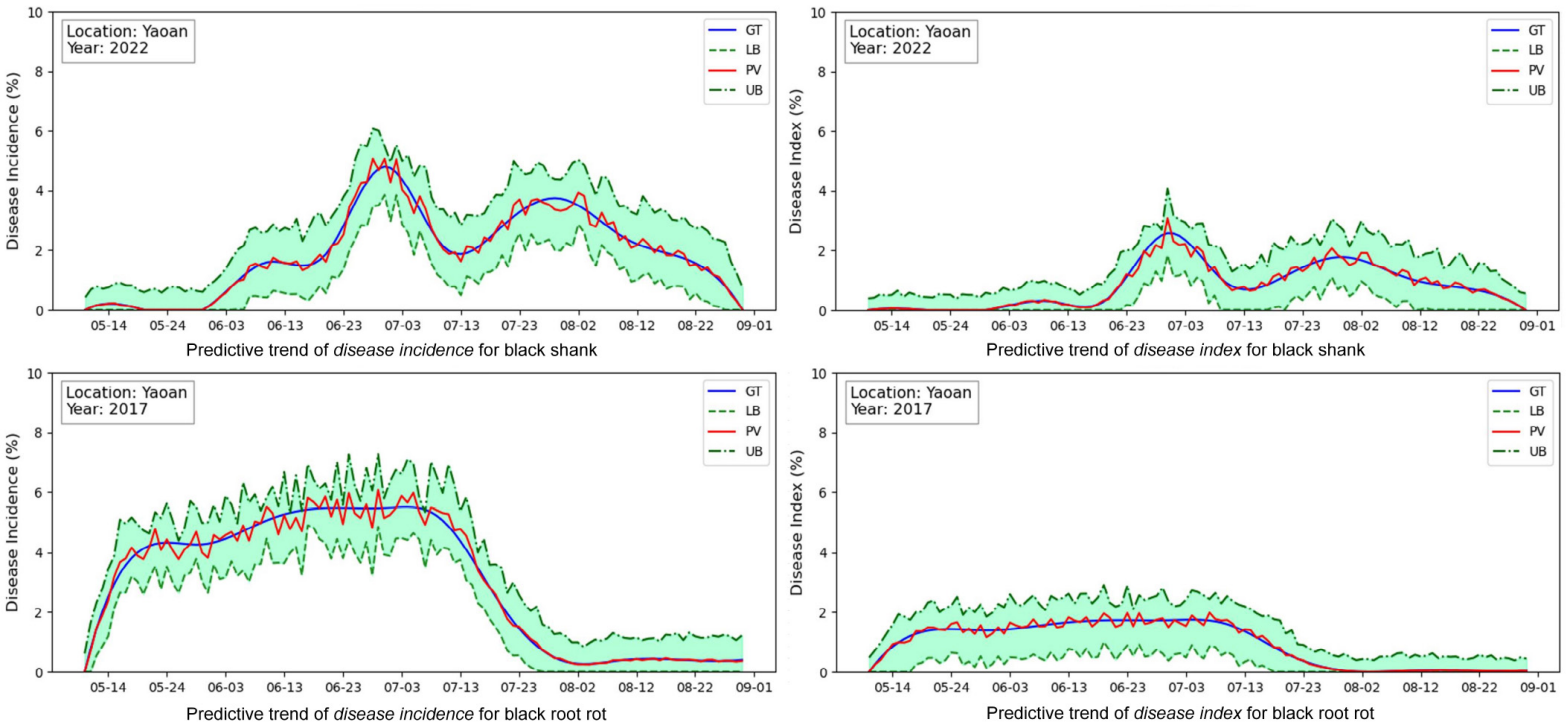
Analysis on predictive trends of the disease incidences and disease indices at the monitoring location (Yaoan). The horizontal axis represents the date. GT, ground truth; PV, predicted value; UB, upper bound, predicted *q*-quantile with *q* = 0.9; LB, lower bound, predicted *q*-quantile with *q* = 0.1.

To better inspect the model prediction capability in predicting disease progression trend, the multi-quantile outputs are conducted to predict the target value $y_{0.5}^{\prime }$ together with the upper limit $y_{0.9}^{\prime }$ and the lower limit $y_{0.1}^{\prime }$ for better inspecting whether the predictive interval covers the ground truth. Figure 4 shows the rolling predictive trends (Left-disease incidences, Right-disease indices) for black shank in 2022 and black root rot in 2017 at Yaoan. The blue and red solid lines respectively denote the ground truths *y* and the predictive values $y_{0.5}^{\prime }$. The upper and lower dashed lines individually indicate the predictive $y_{0.9}^{\prime }$ and $y_{0.1}^{\prime }$. The grass green area indicates the predictive confidence interval.

Firstly, by inspecting the incidences of two diseases on the left in Figure 4, the predictive target values (red solid lines) lightly oscillate around the ground truth values (blue solid lines). This indicates that our model can predict the disease incidences with relatively small errors in most cases. Moreover, the total variation (increase or decrease) trends of red solid lines are similar to the blue solid lines. This proves that our model can predict the overall disease progression trend. Especially, the predictive confidence interval, i.e. the grass green area, most covers the ground truth (blue solid lines) along the time-step. It can provides users with the reliable support information of disease progression even if the point prediction errors occur. Similar conclusions can be drawn on the disease indices on the right in Figure 4. Overall, these results demonstrate our model's fundamental capabilities of trend prediction and reliability estimate (predictive confidence interval), which is enough for management of diseases.

#### 4.3.2 Analysis on independent variable in model input

In this part, in order to provide granular insights into our prediction model, we designed controlled ablation experiments to investigate important determinants supporting the model's prediction efficacy from the following perspectives: independent variables, embedded core modules, and critical training steps.

##### 4.3.2.1 Contribution of historical observations of target variables

The past observations are crucial to forecast. In our forecast model, the historical records of weather variables are easily accessible. However, the past observations of target variables, i.e. the disease incidence and the disease index, involve high acquisition costs. In practice, the records of disease metrics may be non-existent or collected at sparse intervals (e.g., 5-day sampling frequency). In our model, a RPM module was designed to simulate and handle this case. In order to verify its roles, we designed an ablation study as follows.

In this investigation, the different experimental settings, i.e., training and test configurations, are combined to conduct experiments to report results. The training configurations include two scenarios: *without RPM* and *with RPM*. When the RPM is disabled (*without RPM*), all observations of disease incidence and disease index in the past time window are available for each sample. When enabling the RPM (*with RPM*), the observations in the lookback period (e.g., 1-, 3-, 5-, or 10-day relative to the current date) are randomly masked for each sample. In the training process, a model was optimized using the samples with all past observations of disease metrics, and then the model was fine-tuned by the samples with the randomly-masked past observations. In the test, there are five configurations (rightmost 5 columns in Table 4): full past disease observations (no mask) are available or recent several-day observations are masked (unavailable) for each test sample. It is noted that past observations of weather variables are always available and only past observations of disease incidence and index are randomly masked.

**Table 4:** Prediction performance (Avg. MAE ) of disease incidence (%) for two diseases in the different training and test configurations.

**Training setting**	**Disease category**	**No mask**	**1-day mask**	**3-day mask**	**5-day mask**	**10-day mask**
*Without RPM*	Black shank	0.0906	0.0964	0.1047	0.1196	0.1653
	Black root rot	0.0771	0.0827	0.0900	0.1027	0.1423
*With RPM*	Black shank	0.0903	0.0912	0.0929	0.0951	0.1127
	Black root rot	0.0766	0.0774	0.0789	0.0811	0.0959

The smaller the value, the higher the prediction accuracy. The test setting “No mask” indicates full past observation is available. In the test setting “10-day mask,” it means that only weather factors and management information (no past observation of disease metrics) are used for forecasting.

Table 4 shows the comparison MAE results of disease incidences for two diseases in different experimental settings. Firstly, by inspection of results in the setting of “*without RPM*”, we find that the prediction performance in test degrades sharply as the absence of past observations increases when RPM is disabled during training. For instance, for the black shank, the MAE increases from 0.0906 to 0.1653, with 82.47% degradation. This indicates that the model learned in the setting of “*without RPM*” is non-robust to the absence of past observations of disease metrics. When deployed in real-world applications, the absence of partial historical observations of diseases may lead to the drastically-degraded prediction accuracy. The primary cause of this phenomenon is analyzed as follows: During training, when full historical observations of disease incidences are available, the model becomes overly reliant on past disease incidence values (especially for the recent observations) to predict future disease incidences. However, when historical values of disease incidence are missing in test, this reliance will lead to significant prediction bias.

When the RPM is adopted during training, the above performance degradation caused by the absence of historical disease metrics is significantly alleviated. For instance, for black shank, the 24.83% degradation for “*with RPM*” is far lower the 82.47% degradation for “*without RPM*”. This shows the model become robust to missing past observations of disease incidence. This improvement can be explained as follows: The RPM-enabled training exposes the model to diverse samples missing different-period observations of disease incidence. Through this process, the model learns to leverage complete observations of weather variables and past partial observations of disease incidence (even no observations) to predict future disease incidence. In this process, the informative features in the past observations of weather variables will be fully explored and leveraged, and thus the model's capability and robustness are enhanced.

In practical applications, the latest 5-day disease records are frequently unavailable in forecasting. In this case (corresponding to “5-day Mask”), for two diseases, the model using RPM individually achieves 20.48% and 21.03% lower MAE comparing with one without RPM. Especially, even if no historical disease records are available (corresponding to “10-day Mask”), it can only leverage the time-varying weather data and the static management variables to generate relatively reliable forecasting results, e.g., the MAEs of 0.1127 and 0.0959. In summary, these results prove that the RPM enable model learning to explore helpful information from growth conditions and it improves the robustness to the absence of disease records in forecasting.

##### 4.3.2.2 Roles of condition-configurable management factors

In our forecast model, besides time-varying independent variables, the static covariates representing cultivation management information are also significant factors for forecast. Among these static covariates, the disease category, survey year and location are mandatory variables. The others, such as variety, rotation crops, seeding methods, management level and historical disease level in Table 1, are optional (condition-configurable) variables. In this experiment, we conduct an ablation study to analyse the individual contribution of five optional covariates to prediction accuracy. For simplicity, we directly evaluated their contributions by comparing MAEs in some specific test configurations. The baseline configuration excludes all optional covariates. To highlight the contributions of management factors, for the time-varying variables, only the weather data is adopted.

**Figure 5:**
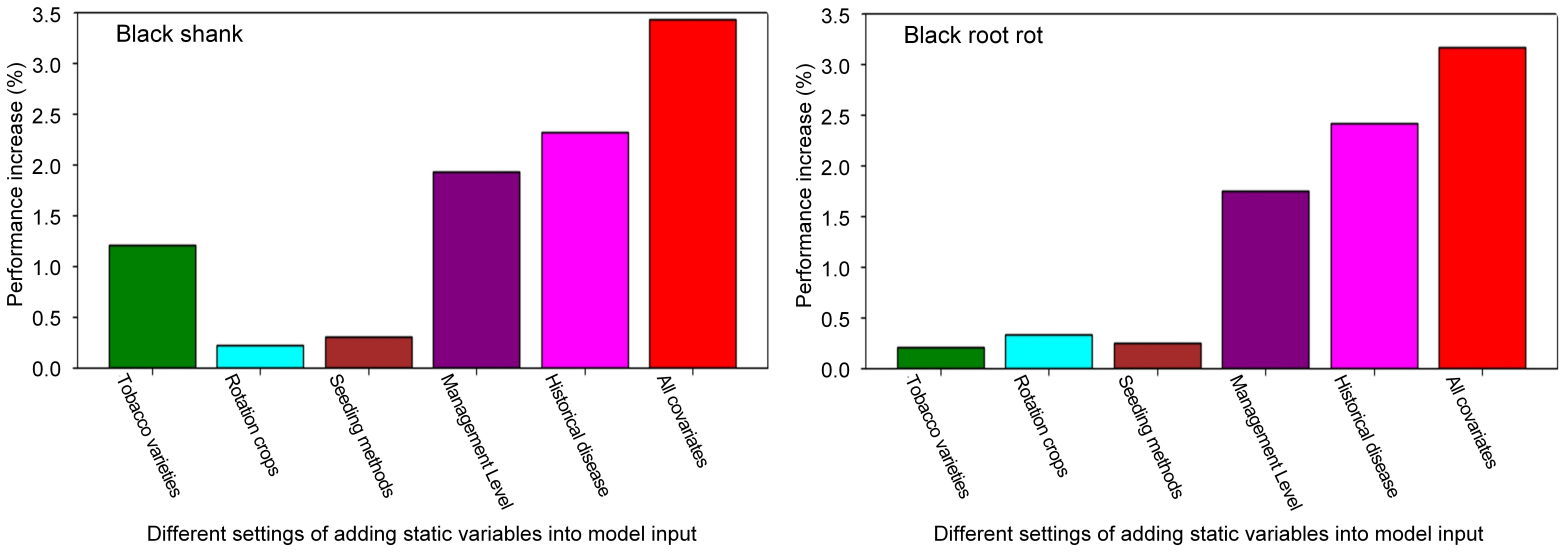
Analysis on static optional covariates for two diseases. The performance increase (%) is evaluated by the MAE relative reduction compared to the baseline. The last bar denotes all covariates are together added. For the others, each bar indicates a covariate is individually added.

The results in Figure 5 demonstrate the individual contribution of each optional covariate for two diseases. Two largest MAE reductions relative to the baseline are individually achieved by the management level or the historical disease level. This demonstrates their substantial contributions to enhancing forecast performance. Through similar comparative analysis, we identified that the variety observably enhances the prediction accuracy for black shank, but shows limited utility for black root rot. This primarily stems from that our model learns the knowledge that disease-resistant varieties suppress black shank to some extent. Conversely, both seedlings and rotation crops demonstrated a tiny contribution for two diseases. This is mainly because there remains substantial uncertainty in their association with disease risks, making it challenging for models to extract statistically significant patterns from the data. Finally, two right red bars show that more informative features can be explored for two diseases when integrating all optional covariates.

##### 4.3.2.3 Cross-temporal analysis on weather factors

Besides cultivation management, weather conditions serve as deterministic factors in both the initiation and exacerbation of disease epidemics. In this experiment, we analyze how the model utilize historical observations of weather variables to predict disease risk and progression trends, including initial outbreak, exacerbation and mitigation. To this end, a sample-wise inverse inference and analysis approach was conducted to determine which weather variables and what cross-temporal variations principally contribute to the prediction result. Especially, to highlight the contributions of weather variables to prediction results, in this experiment, observations of disease metrics were excluded; only weather data were used for forecasting.

The interpretable feature extraction in our model is employed to implement the inverse inference and analysis approach. The operational procedures are described as follows: (1) Some representative samples characterizing the initial outbreak, exacerbation, and mitigation phases of a disease were selected. (2) An sample was fed into the model to conduct prediction and evaluate what contribute to the predicted result by examining the feature derivation mechanism. Firstly, in the GTFF module, according to the method quantizing the importance of time-step (Lim et al., 2021), we evaluated the attention weights in Equation 7 to compute a time-step-wise weight α_*k*_. A 10-dimensional vector [α_1_, ⋯ , α_10_] was obtained for past 10 days. Then, in each time-step, a 6-dimensional weight vector [ω_1_, ⋯ , ω_6_] for weather variables can be extracted from the model as shown by the VSFN in Figure 3. Finally, by combining two kinds of weight vectors, we can obtain a weight matrix *M*∈ℝ^6 × 10^ cross the weather dimension and temporal dimension. After normalization, its heatmap was employed to express the cross-temporal importance of weather variables.

**Figure 6:**
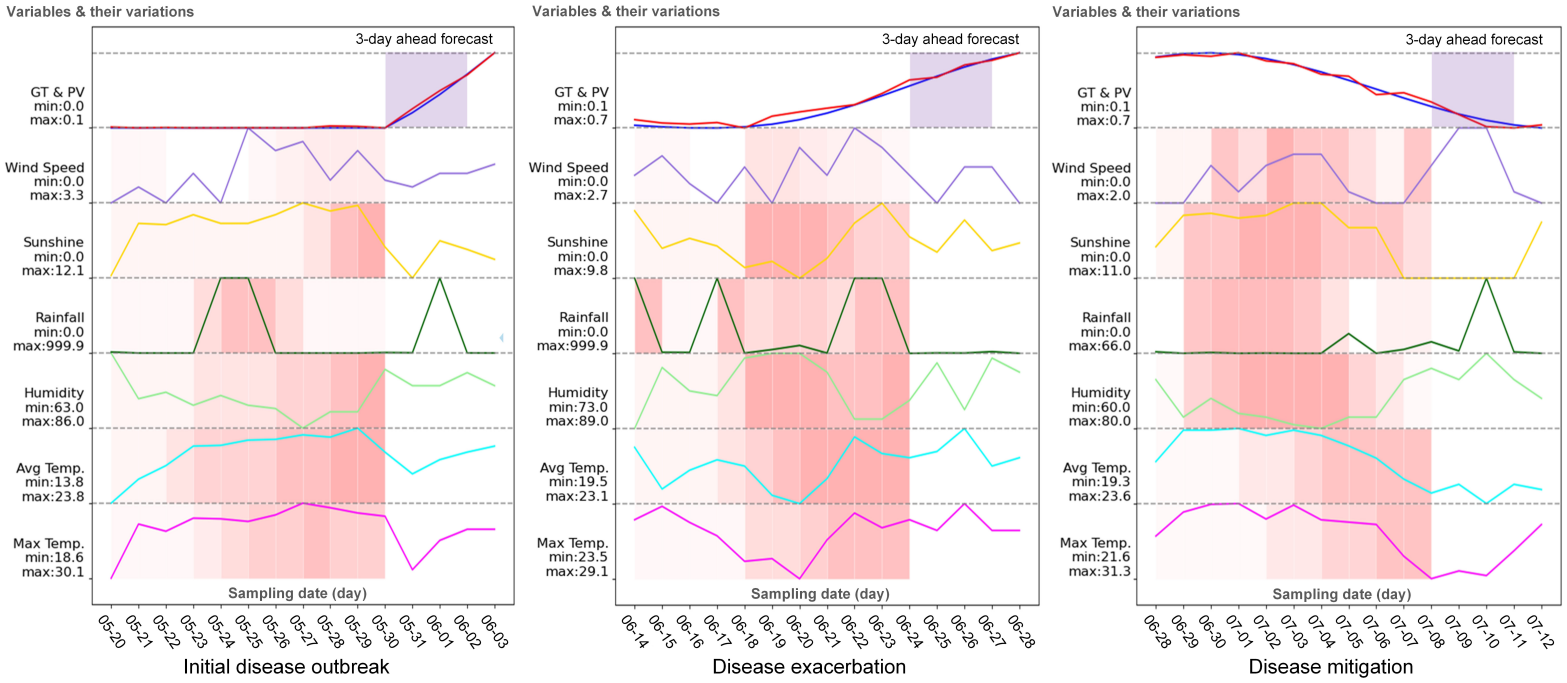
Analysis on feature patterns of time-series weather variables in predicting the disease incidence of black shank at Lufeng in 2004. GT-Ground Truth for disease incidence (blue solid line); PV-Predicted Values (red solid line). For the heatmap, darker red indicates that the historical observation of variable has a greater contribution to disease development events (disease outbreak, disease exacerbation and disease mitigation) in the light purple regions.

Figure 6 presents analytical results under three characteristic scenarios: initial disease outbreak, disease exacerbation and disease mitigation for black shank. As shown by the left chart in Figure 6, the multi-day rainfall around May 25th, the following conditions such as high temperature and high humidity, and the bad ventilating are the main inducing factors for the occurrence of black shank. Similarly, we can find that the high daily-average temperature and the suitable humidity contribute much to the disease exacerbation as shown by the middle chart in Figure 6. However, after analyzing more than 20 samples with the decreasing disease incidences, we did not find the common statistical characteristics of weather variables for disease mitigation. Only a few samples prove that the multi-day low-humidity and long sunshine duration suppress the pathogenic bacteria, as shown by the right chart in Figure 6. The main reason is because the disease mitigation more relies on the manual management control, e.g., spaying farm chemicals. The interaction between pathogen dissemination and pesticide suppression is an intricate and highly uncertain process.

## 5 Discussion

**Figure 7:**
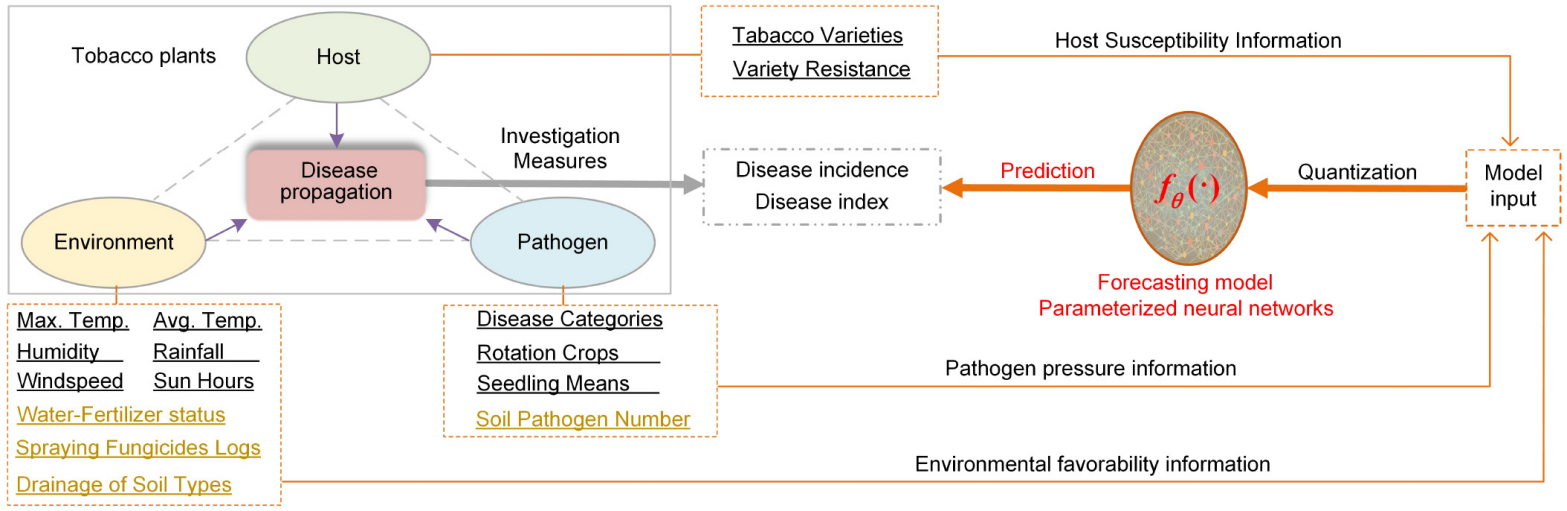
Discussion on forecast model in the host-pathogen-environment disease triangle framework. The model *f*_θ_(·) simulates the disease propagation process to predict the disease incidences and disease indices based on the sampled information from host, pathogen and environment. The black underlined text denotes the independent variables adopted in this work. The khaki underlined text indicates the elaborate factors which can be extended.

The forecast model's capability and reliability level mainly depends on the model complexity and input data quality. In disease management, the three-component system of host susceptibility, pathogen pressure and environmental favorability governs infection risks as shown in Figure 7. To accurately forecast disease risks, we should model the relation between disease propagation and the independent variables in each component. Meanwhile, the model should be able to characterize the interaction between three components. Next, we will discuss our model in the context of disease triangle framework (Scholthof, 2007), from the multiple aspects, i.e., model input, model complexity and capability, and computational cost.

Firstly, the flexible and hybrid-structure model input *X* = {*X*_*H*_, *X*_*F*_, *X*_*S*_} integrates multi-aspect factors of pathogen, host and environment as show in Figure 7. Our data-driven time-series forecasting model well describes the dynamic characteristics of independent variables in three components and learn how different factors affect disease progress. When more helpful variables are measured and added in the model input, the forecasting accuracy can be further improved. Moreover, our PHFTNet-RPM has scalable model complexity and capability. Relying on the condition-configurable input and the model's inner modules with adaptive expressive-capability, the model can dynamically adjust prediction functionality in response to diverse and complex inputs. Especially, when the dimension of model input and the data scale increase, the models capacity can be scaled via hyperparameter tuning, for example, enhancing the hidden state size *n*_*e*_. However, incorporating field management variables into the model or enhancing model complexity through parameter tuning will inevitably lead to increased computational costs.

In comparison with the existing disease forecasting methods (Wang et al., 2024; Chen et al., 2024, 2025), the key improvements of our method are summarized as follows: (1) Field management variables, key factors in disease development, are integrated to improve feature learning in the forecasting model. (2) The RPM module is tailored to handle incomplete or delayed disease data. It effectively prevents performance degradation during testing when disease data is missing. (3) A well-designed PHTFNet-RPM can effectively integrate heterogeneous inputs and learn cross-variable, cross-temporal feature representations. (4) The uncertainty estimate is incorporated in the model. It not only enhances the model's robustness but also helps agronomists make scientifically-informed management decisions.

## 6 Conclusion

In conclusion, this paper proposed a solution for forecasting risks of tobacco root diseases such as black shank and black root rot. The extensive experiments prove this solution is effective and reliable. The core of solution relies on the proposed PHTFNet-RPM forecast model based on deep neural networks. The carefully-designed internal modules and scalable network architecture endow the model with a flexible and powerful representational capability. Through time-series feature learning, it can acquire knowledge from training data such as historical weather records, disease observations, and field management information, achieving accurate prediction of disease risk trends. Moreover, thanks to its flexible hybrid-structured input and adaptive model expressiveness, users can configure the prediction model according to the availability of field management data and historical disease observations, allowing for condition-configurable adaptive forecasting. In fact, our model can even achieve relatively accurate disease trend predictions based solely on historical weather data.

Furthermore, to enhance the practicality of the model, the uncertainty estimation based on probatilistic prediction and Bayesian learning theories is introduced into both model design and training. It allows for direct evaluation of confidence intervals in predictions, thereby improving the trustworthiness of prediction results. Moreover, during real-world deployment and application, the reliability of model can be assessed by randomly selecting samples from specific scenarios, which helps improve its robustness across various real-world conditions. Specifically, our disease forecasting approach is based on a data-driven learning model with scalable representational capability. If relevant training data is available, it can be extended not only to forecast other tobacco diseases, but even to predict diseases for other crops as well.

In the future, we will continue research efforts focusing on improving the accuracy of disease forecasting and expanding the application scenarios of the model: (1) Integrate future weather forecast data into the models input to improve prediction accuracy by providing more available helpful information. (2) Deploy the model in real-world settings for testing and evaluation, and collect more training data to fine-tune the model with newly-acquired datasets, continuously enhancing its reliability and practicality. (3) Gather more detailed environmental and management data from modern tobacco farms, e.g. time-continuous water-fertilizer status and pesticide usage logs in tobacco field growth stage, and incorporate them into the model input and train the model, enabling it to learn forecasting knowledge tailored for the small-scale, high-reliability scenarios.

## Data Availability

The datasets presented in this study can be found in online repositories. The names of the repository/repositories and accession number(s) can be found below: https://github.com/85723015/TobaccoDiseaseForecast.

## Footnotes

1
https://github.com/sktime/pytorch-forecasting
